# Nutrient availability and pH level affect germination traits and seedling development of *Conyza canadensis*

**DOI:** 10.1038/s41598-021-95164-7

**Published:** 2021-08-02

**Authors:** Caroline Maldaner Follmer, Ana Paula Hummes, Nadia Canali Lângaro, Claudia Petry, Diovane Freire Moterle, Edson Campanhola Bortoluzzi

**Affiliations:** 1 University of Passo Fundo, Campus I, BR 285, km 292, Passo Fundo, Rio Grande do Sul, 99052-900 Brazil; 2 Postgraduate Program in Agronomy, University of Passo Fundo, Campus I, BR 285, km 292, Passo Fundo, Rio Grande do Sul, 99052-900 Brazil; 3 Federal Institute of Education, Science and Technology of Rio Grande Do Sul, Osvaldo Aranha, Bento Gonçalves, Rio Grande do Sul, 540, 995700-000 Brazil; 4 Laboratory of Land Use and Natural Resources, University of Passo Fundo, Campus I, BR 285, km 292, Passo Fundo, Rio Grande do Sul, 99052-900 Brazil

**Keywords:** Environmental chemistry, Environmental impact, Biogeochemistry, Environmental sciences

## Abstract

Reducing pesticide application in agricultural land is a major challenge for the twenty-first century. Responses of weed seed’s germination and seedling’s early development to chemical soil conditions around the seed may be a promising way to aid weed control in a no-till system. Thus, the objective of this work was to test, under controlled conditions, whether different chemical conditions affect the germination and development of horseweed [*Conyza canadensis* (L.) Cronquist]. We used, as treatment, solutions containing different nutrients (P, K, Ca, and Mg), separately and in combination, and at two pH levels (4.8 and 6.5). Phosphorus alone inhibited horseweed seed germination at ~ 7 times while had ~ 4 times reduction in final germination percentage and germination speed index for both pH tested. Other nutrients tested had a no-effect in germination speed index compared to the control treatment. Potassium alone or associated with other ions (P, Ca, and Mg) at pH 4.8 had a synergistic effect on seedling development (root and shoot length). In the same way, K associated with Mg was synergistic to the root length at pH 6.5. Seeds in the control treatment (distilled water) presented a high germination speed index at pH 6.5, while at low pH this parameter was higher when in association with KMg, PMg and Ca. The findings demonstrate that seed germination traits and seedling development of horseweed depend on nutrient kind exposure and pH conditions in the seed environment. This work suggests that adequate topsoil management (i.e., pH and nutrient availability) may aid to reduce weed germination, because, it consists of an important factor of weed occurrence in agricultural areas.

## Introduction

The reduction of pesticide application is a major challenge in modern agriculture for the twenty-first century. No-till system (NT) implemented in the 1970s decade promoted better soil quality since soil cover, crop rotation, low soil disturbance, adequate traffic load, and adequate dose and fertilization method were practiced^1^. However, when these aspects are mismanaged, agricultural areas are subject to soil degradation^2^, low productivity and weed occurrence^3^, increasing pesticide uses^4^, and environmental risks^5^. Weeds present high seed production, dissemination, and cropland infestation capacities, causing mainly yield reduction. The species ability to complete germination and grow in a large range of edaphic conditions engender competitive advantages to the weeds over crops^6^. It is a key mechanism of success in plant establishment in different environments^7^, but the seed germination process can be affected by several factors such as light regime, temperature, and soil moisture^8^.

From an agronomic perspective, the genus *Conyza* Less. is responsible for decreasing soybean yields up to 30%^9,10^. The species of this genus are easily found in no-till system, where they became a great issue in the cereal crop production worldwide^3,8^. Recently, agronomists have found *Conyza* spp. biotypes with herbicide resistance mechanisms to several herbicide molecules, including glyphosate^4,11^. In South Brazil, 78% of the *Conyza* spp. biotypes showed herbicide resistance to glyphosate^4^. In summary, a good chemical horseweed control became arduous^4,12,13^. Furthermore, because this species is stress-tolerant and strong competitors^10,14^, alternative management strategies should be considered in the no-till system. Those strategies based on the species biology/ecology and soil management applied in an integrated manner seem to be promising to reduce the pollution pressure on the environment^8,15,16^. In this sense, *Conyza* sp., a dicot weed of the Asteraceae family commonly known as horseweed, stands out among weed species because can produce large quantities of easily disseminated seeds^8,15^. Germination is stimulated by light and may occur on bare topsoil in all seasons^8,17,18^. The wind may carry horseweed seeds over a hundred meters^15^, while seeds can migrate from infested fields to new areas a few thousand kilometers distant attached to cattle hair^19^. Horseweed seeds reach cropland topsoil easily, enriching the soil seed bank^8^. The horseweed seeds in the soil seed bank may germinate under favorable conditions from few years to decades^8^. The horseweed germination may occur by neutral to alkaline pH, saline and not disturb soil conditions, but maybe disturbed in the presence of Aluminum (Al) and when temperature and humidity conditions are out of the ideal range^3,8,18,20,21^. Furthermore, the species respond to factors such as salinity (NaCl)^7^, nutrient availability (N, P, and K), elements’ toxicity, and soil pH^7,22–25^. Thus, we expect that other nutrients available in soil may favor weed establishment and their dominance^26,27^.

These studies suggested then that different cations affect the germination process. However, scarce literature reports on germination of horseweed at acid conditions (low pH) and in presence of macronutrients found commonly in fertilizer and lime products^28^. This is particularly important to the no-till system, which has been receiving lime and fertilizer products on the topsoil, promoting a nutrient enrichment a few centimeters of soil surface. This scenario was not yet been studied face to the *Conyza canadensis* (L.) Cronquist var. canadensis germination and development. Thus, we expected that horseweed seeds may have a variable germination percentage and seedling establishment caused by exposure to different nutrients and pH levels, as suggested in the literature for other species^7,25,26,29,30^. In this study, we pay attention to two basic questions: which are the nutrients and pH level that act preponderantly in the germination and development of horseweed? and; what are the implications for horseweed management and fertilization practices on the field conditions? In this direction, studies on the factors involved in horseweed germination and establishment may allow improvement in management practices to avoid weed infestations in no-till system, reducing pesticide products as a control agent. We hypothesize that seed exposure to different nutrients and pH levels affects seed germination traits and seedling development. We expected also that eutrophic conditions present three possibilities of effects: synergistic, antagonistic, or no-effect. Identifying whether nutrients drive *C. canadensis* germination and plant establishment is essential to provide a better knowledge on fertilization in agricultural areas to help weed control and diminish pesticide application.

Thus, this study aimed to evaluate the seed germination traits and seedling development of horseweed in different chemical environments with a range of nutrient availability and pH conditions. For this, horseweed seeds were exposed to several nutrients (P, K, Ca, and Mg, alone and in combination) under two pH levels (4.8 and 6.5). The nutrients and their concentrations as well as the pH conditions were chosen to simulate field conditions of fertilization and liming commonly practiced in soybean crop under no-till system in south Brazil.

## Results

### Overview on anova

The nutrients, as a factor of variation (F1 Factor), affected significantly all the parameters evaluated (Table 1). The pH (F2 factor) did not affect horseweed’s final germination percentage and germination speed index and root length, except for seedling length and root:shoot ratio. The interaction between nutrients and pH (F1 vs F2) affected all variables.

### Final germination percentage (FGP) and nutrients

**Figure 1:**
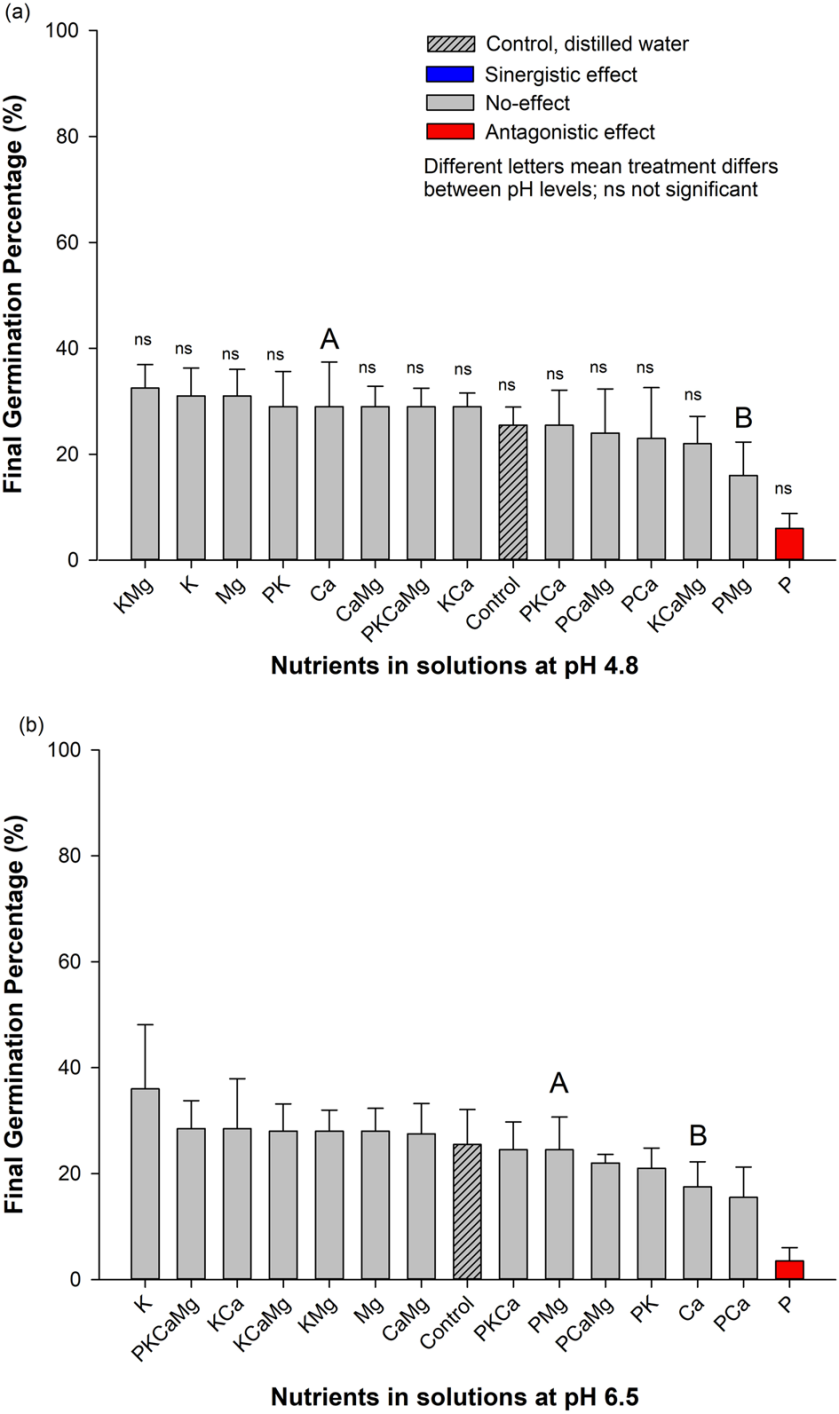
Final germination percentage (FGP) of *Conyza canadensis* in response to the nutrients (alone or in combination) under solutions at pH 4.8 (**a**) and at pH 6.5 (**b**). Bars with the different colours, comparing means among treatments, are significantly different, according to the Tukey’ test (*p* < 0.05). Different letters represent a statistical difference for the same treatment between the two pH tested while *ns* represents not significant; The results in the Bars represent means, while error bar marks represent standard deviation (*n* = 4) from the mean value.

Different nutrients affected the FGP by the average of both pH levels (Fig. 1a,b). Two effects were observed (i.e., no-effect and antagonistic effect). In both pH (4.8 and 6.5), two effects were observed (i.e., no-effect and antagonistic effect) on FGP. At low pH (pH 4.8), nutrients alone or in combination did not differ from the control treatment (FGP of 25.5%), except for the treatment with P that presented the lowest *C. canadensis* final germination percentage (6%) (Fig. 1a). This indicates an antagonistic effect of P (4.25 times) on the final germination percentage compared with the control. At high pH (6.5), the treatment with P alone presented the lowest value, 3.5% (Fig. 1b). As the treatment had a reduction in germination around 7.28 times compared to the control, they had an antagonistic effect. In addition, all other nutrients alone or in combination had no-effect because did not differ from the control (distilled water).

### Final germination percentage and pH levels (acidity)

The pH alone did not influence final germination percentages. On average, the FGP was not sensitive to the pH levels and their values were similar, 25.3% and 26.8% to high and low pH tested, respectively. Furthermore, the pH presented interaction with only Ca and PMg treatments. The final germination percentage was higher for Ca (29%) at low pH than at pH 6.5 (17.5%), while PMg treatment was higher at pH 6.5 (24.5%) than at pH 4.8 (16%) (Fig. 1a,b).

In summary, P alone inhibited horseweed seed germination in 4.25 and 7.28 for both pH tested, 4.8 and 6.5.

### Germination speed index (GSI) and nutrients

**Figure 2:**
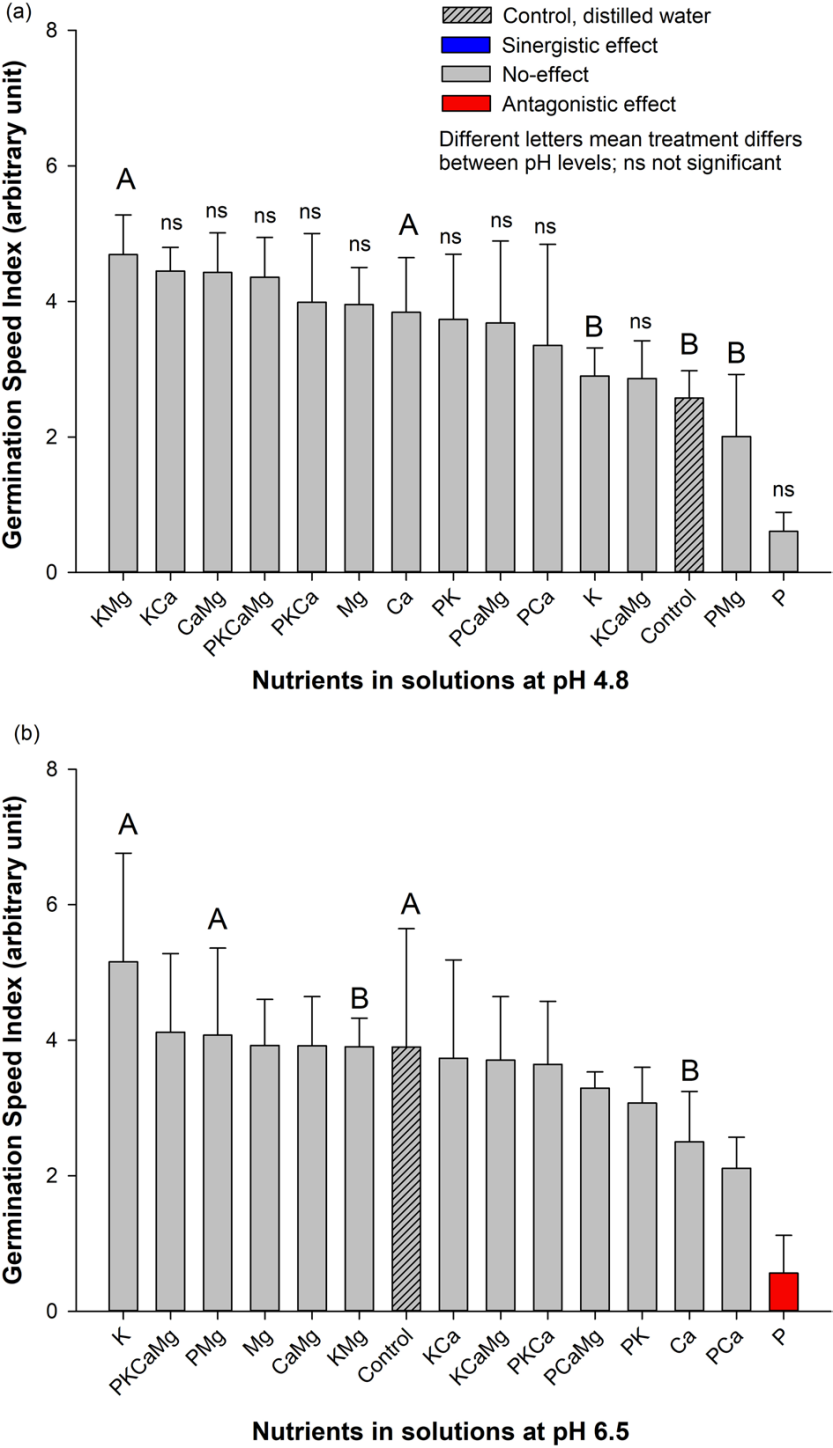
Germination speed index (GSI) of *Conyza canadensis* in response to the nutrients (alone or in combination) under solutions at pH 4.8 (**a**) and at pH 6.5 (**b**). Bars with the different colours, comparing means among treatments, are significantly different, according to the Tukey’ test (*p* < 0.05). Different letters represent a statistical difference for the same treatment between the two pH tested while *ns* represents not significant; The results in the Bars represent means, while error bar marks represent standard deviation (*n* = 4) from the mean value.

For germination speed index (GSI), different nutrients had different effects at each pH level (Fig. 2a,b). At low pH (4.8), nutrients produced no-effects on the GSI (Fig. 2a). At high pH (6.5), two effects were observed (i.e., no-effect and antagonistic effect) (Fig. 2b). All treatments (Ca, Mg, K, CaMg, PCa, KCa, KMg, KCaMg, PCaMg, PKCa, PKCaMg, and, PCa) presented similar GSI values to the control treatment, indicating no-effect of nutrients compared to the control. Phosphorus alone presented the lowest GSI value (0.56, au), indicating an antagonistic effect comparatively to the control. The GSI reduction was 6.96 times in the GSI, comparatively to the control.

### Germination speed index and pH levels

In the average of all nutrients, the GSI was not affected following the pH levels. In addition, pH interacted with KMg, Ca, PMg, K, and control treatment. At pH 4.8, the GSI values were higher for the KMg (4.69), Ca (3.8), K (5.2) presenting 1.20, 1.53, 1.78, and 1.34 times greater than pH 6.5, respectively. At pH 6.5, PMg (4.08) and control (3.9) treatments were higher compared to the pH 4.8, presenting 2.02 and 1.51 times greater, respectively.

Briefly, phosphorus alone had an antagonist effect, reducing GSI in both pH conditions by an average of ~ 5.6 times (Fig. 2).

### Seedling length (SL) and nutrients

**Figure 3:**
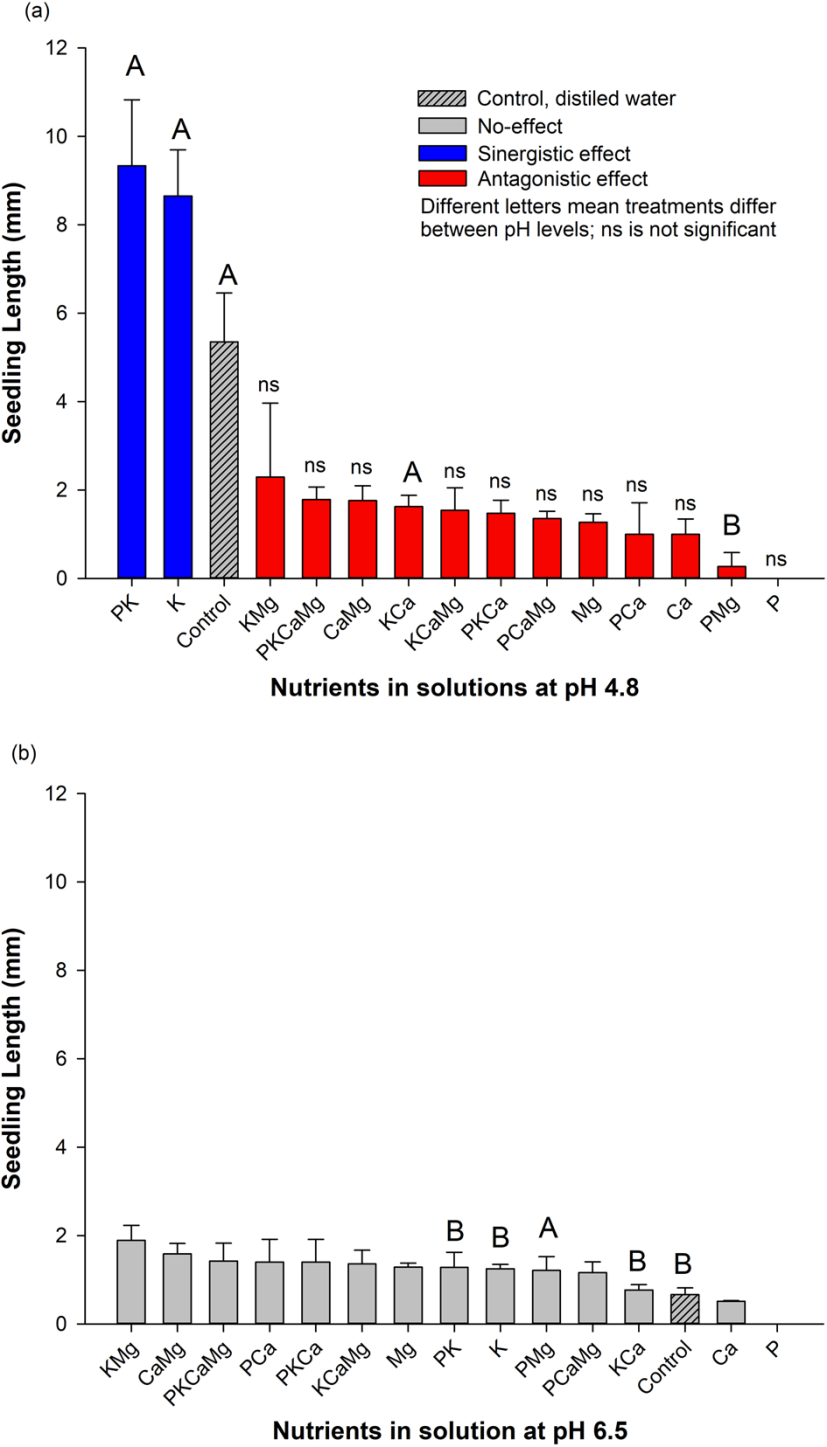
Seedling length in aerial part (SL) of *Conyza canadensis* in response to the nutrients (alone or in combination) under solutions at pH 4.8 (**a**) and at pH 6.5 (**b**). Bars with the different colours, comparing means among treatments, are significantly different, according to the Tukey’ test (*p* < 0.05). Different letters represent a statistical difference for the same treatment between the two pH tested while *ns* represents not significant; The results in the Bars represent means, while error bar marks represent standard deviation (*n* = 4) from the mean value.

For SL, different nutrients had different effects follow the pH levels (Fig. 3a,b). At low pH (4.8), nutrients produced three effects (i.e., synergistic, no-effect, and antagonistic effect) (Fig. 3a). The PK combination and K alone presented the highest SL values (9.34 mm and 8.65 mm, respectively), comparatively with the control (5.35 mm). This indicated a synergistic effect of PK and K nutrients on SL compared to the control, increasing the values in 1.74 and 1.62 times, respectively. All other nutrients (Mg, Ca, K, PK, PCa, PMg, PKCa and, PCaMg, KMg, KCa, CaMg and PKCaMg) differed from the control, indicating an antagonistic effect. Phosphorus and PMg presented the lowest SL values, representing a 19.8 times reduction compared to the control treatment. At high pH (6.5), no-effect was observed among the treatments compared with the control (Fig. 2b). All treatments presented similar SL values among them.

### Seedling length and pH levels

The pH had an interaction with some nutrients (Fig. 3). On average, the SL at low pH (2.76 mm) was higher than at pH 6.5 (1.22 mm). Comparing SL between both pH, the treatments control, PK, K and, KCa presented 8.05, 7.30, 6.93 and, 2.12 times greater at pH 4.8 than at pH 6.5, respectively. In opposition, the PMg treatment presented 4.47 times greater SL at pH 6.5 than at pH 4.8 (Fig. 3).

Briefly, K alone or in combination with P was synergistic for shoot length (SL) at low pH, while all other combinations were antagonistic at low pH. At pH 6.5, a wide range of combinations of nutrients ad no-effect on SL.

### Root length (RL) and nutrients

**Figure 4:**
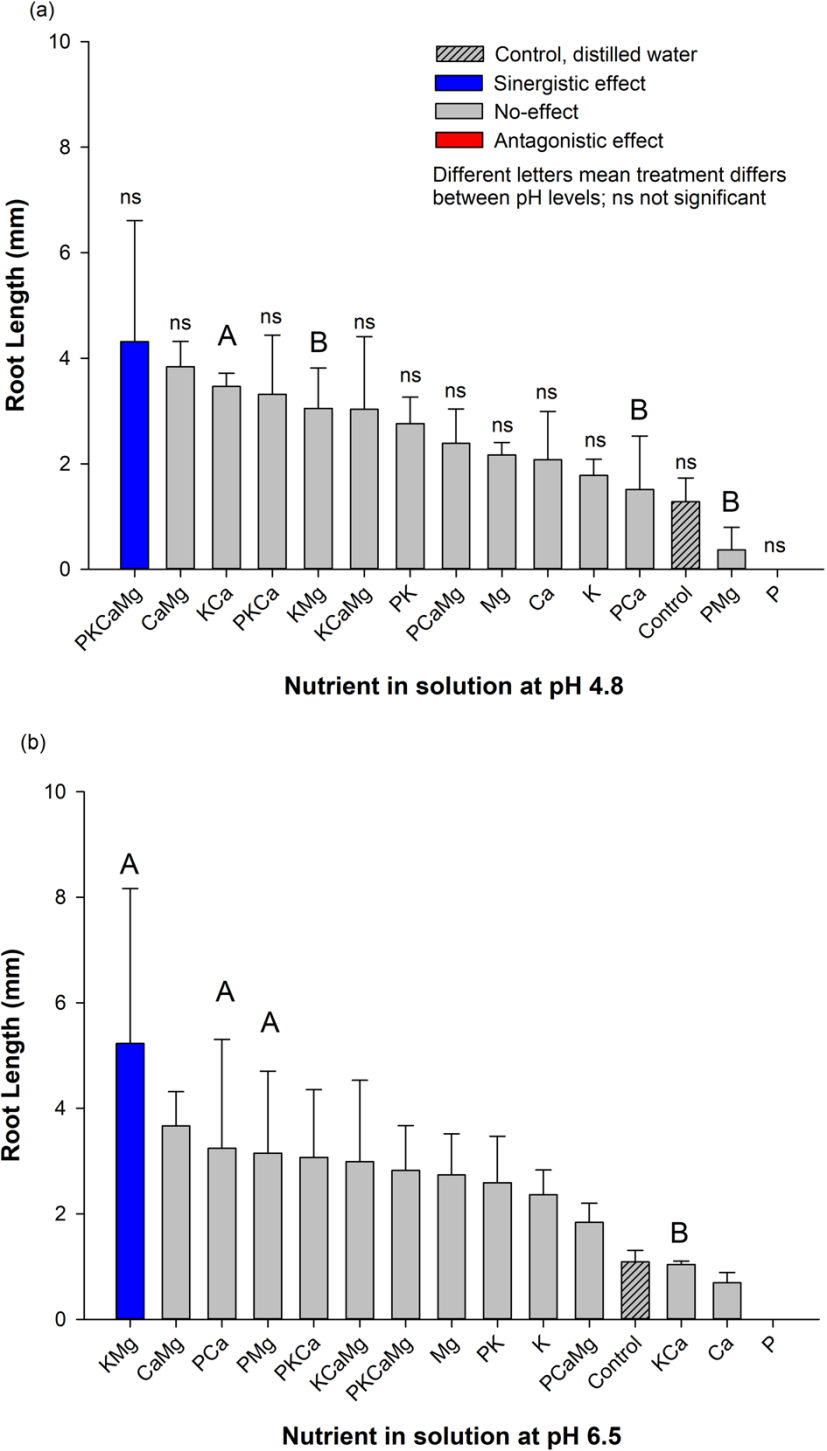
Root length (RL) of *Conyza canadensis* in response to the nutrients (alone or in combination) under solutions at pH 4.8 (**a**) and at pH 6.5 (**b**). Bars with the different colours, comparing means among treatments, are significantly different, according to the Tukey’ test (p < 0.05). Different letters represent a statistical difference for the same treatment between the two pH tested while *ns* represents not significant; The results in the Bars represent means, while error bar marks represent standard deviation (*n* = 4) from the mean value.

The different nutrients affected the parameter in each pH level (Fig. 4a,b). In both pH (i.e., low and high), two effects on RL were observed (i.e., synergistic and no-effect). At low pH (4.8), the PKCaMg treatment (combination from all nutrient tested) presented the highest RL value (4.32 mm) comparing to the control (1.28 mm), indicating a synergistic effect of nutrients. Furthermore, all other treatments: P, Mg, Ca, K, PK, PCa, PMg, KMg, KCaMg and, PCaMg presented similar SL values to the control, indicating no-effect of these nutrients (Fig. 4a). At high pH (6.5), the treatment KMg presented the highest RL value (5.23 mm), comparing to the control (1.09 mm), indicating a synergistic effect of nutrients (Fig. 4b). The KMg treatment presented RL 4.84 times greater than the control. All other nutrients: alone or in combination presented RL values similar to the control, indicating no-effect of nutrients.

### Root length and pH levels

The pH presented an interaction with nutrients. KCa treatment, presenting higher RL values (3.32, times, respectively) greater at pH 4.8 than at pH 6.5. The KMg, PCa and PMg treatments presented higher RL values (1.72, 2.14, 8,72 times, respectively) greater at pH 6.5 than at pH 4.8.

### Root:shoot ratio (RSR) and nutrients

**Figure 5:**
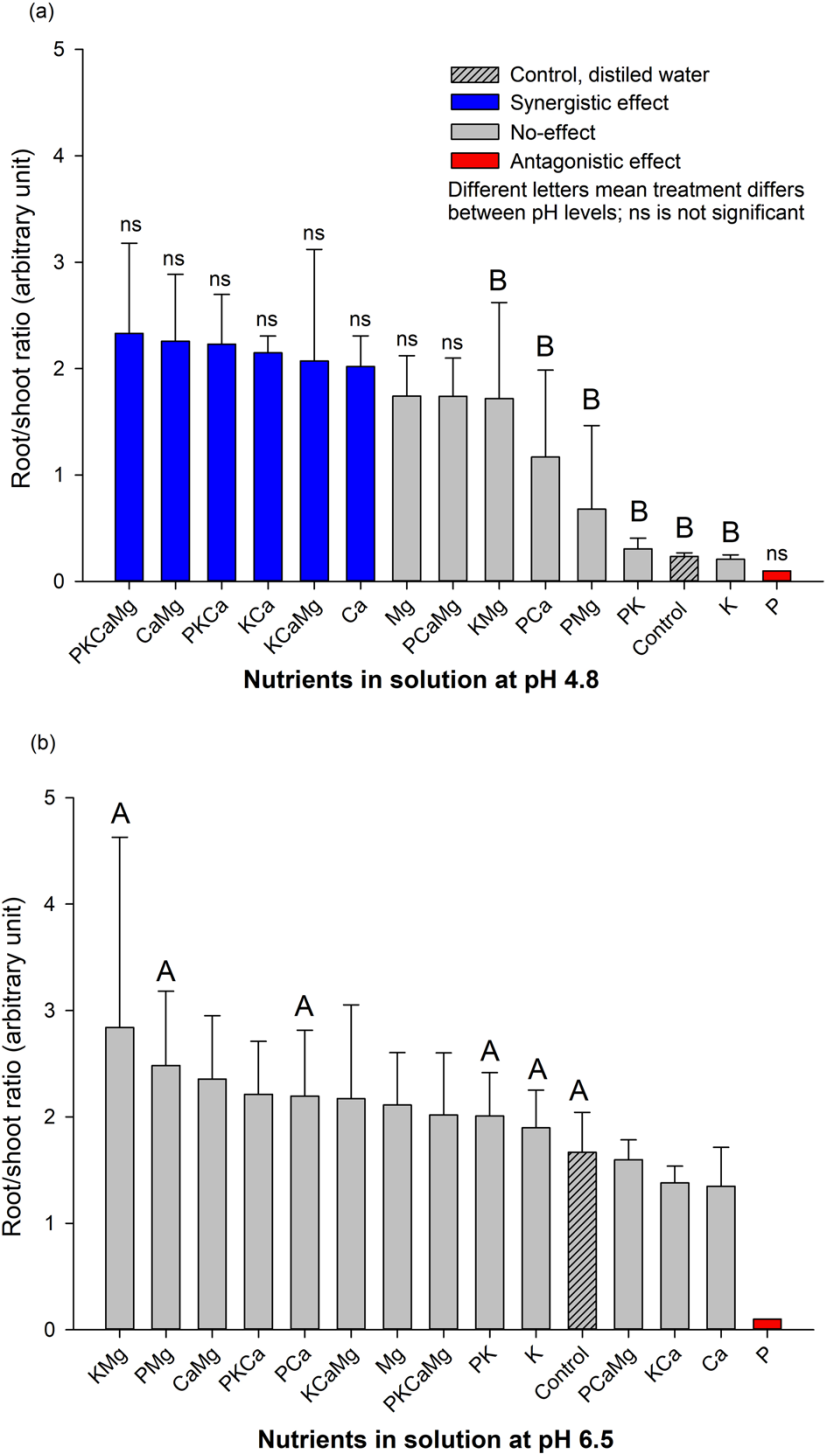
Root:seedling ratio (RSR) of *Conyza canadensis* in response to the nutrients (alone or in combination) under solutions at pH 4.8 (**a**) and at pH 6.5 (**b**). Bars with the different colours, comparing means among treatments, are significantly different, according to the Tukey’ test (*p* < 0.05). Different letters represent a statistical difference for the same treatment between the two pH tested while *ns* represents not significant; The results in the Bars represent means, while error bar marks represent standard deviation (*n* = 4) from the mean value.

At low pH (4.8), nutrients produced three effects (i.e., synergistic, no-effect, and antagonistic effect) on RSR (Fig. 5a). The treatments PCa, PKCa, PCaMg, KCa, CaMg, and PKCaMg presented high RSR indicating a synergistic effect comparing with the control. Phosphorus presented the lowest RSR value, characterized as an antagonistic effect compared to the control treatment. All other treatment presented no-effect because did not differ to the control (5.35 mm). At high pH (6.5), no-effect was observed among the treatments (Fig. 2b), except for the P treatment that demonstrates an antagonistic effect compared with the control.

### Root:shoot ratio and pH levels

The high pH (6.5) presented higher RSR values for KMg, PMg, PCa, PK and, K and control treatments compared with the same nutrients at low pH (Fig. 5b).

### The principal component analysis (PCA)

**Figure 6:**
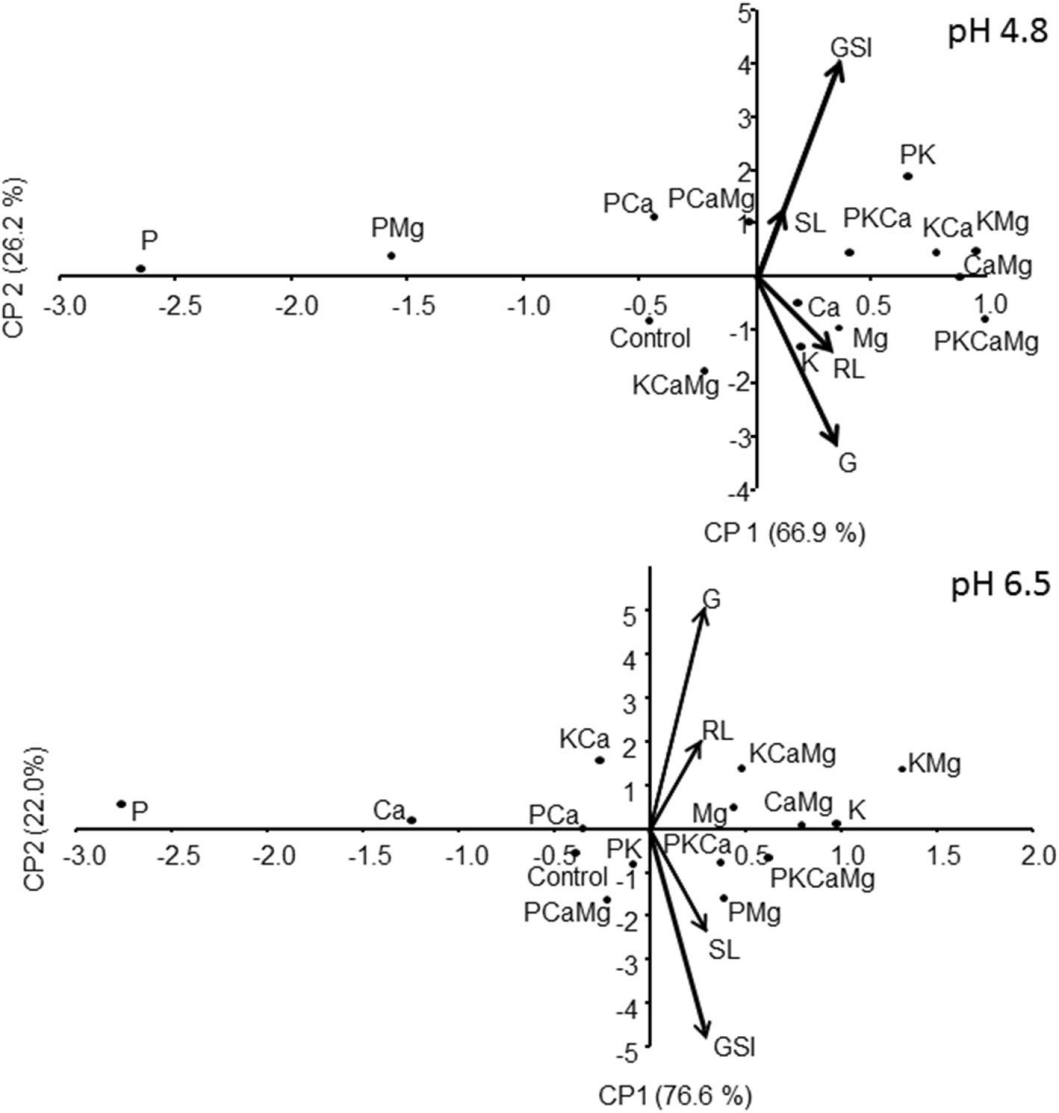
Principal component analyses considering nutrients (alone and in combination) under solution at pH 4.8 and 6.5. G = Final germination percentage; GSI = germination speed index; SL = seedling length in their aerial part; RL = root length; Data set used for each pH level (n = 60; treatments × replications). The principal components (i.e., axes PC1 and PC2) explain the magnitudes in percentage of data variability.

The PCA represented the association degree among studied variables. The first and the second principal components of PCA explained 66.9% and 26.2% of the data variations, for pH 4.8; and 76.6% and 22.0% for pH 6.5 (Fig. 6). Principal component analysis pointed that frequently K alone or in combination with Ca and Mg had a high positive association (vectors in the same directions) with seed final germination percentage, and root length and shoot length of *C. canadensis*. However, P resulted in an antagonistic response for these variables (vectors in opposite directions). Phosphorus associated with the K or Mg was the most prominent nutrients generating contrasting plant responses for germination speed index and seedling development (shoot length) depending on the pH level. Phosphorus showed a greater antagonistic effect, which was a low seed germination percentage; however, P allows a good development of *C. canadensis* seedling. Potassium had a positive correlation with the final germination percentage and root length of *C. canadensis* seeds, especially under low pH.

## Discussion

### Nutrients and pH as factors to seed germination traits and horseweed seedling development

Overall, the studied species had important requirements to germinate, considering the germination traits (FGP and GSI) verified in the control treatment (~ 36%), suggesting an average ability to complete germination. In the same way, the seedlings may develop in a wide range of chemical conditions (pH and nutrients). Then, our study reinforces the literature which this species can easily get establish itself in several topsoil conditions^8^. Two agronomic/environmental reasons can be emphasized as major issues concerning the horseweed management: i) this species has a low control in no-till system and has a great capacity to reduce crop yield^3,8^ and, ii) the no-till system has been receiving annually large amounts of fertilizers (nutrients) and lime on topsoil^28^, regardless the consequences for the weeds’ communities. The present work argues on the early plant development stage (germination and seedling development) because it seems to be a crucial plant stage for better understand the occurrence and establishment of *C. canadensis* in cropland areas. The results evidenced that nutrients, alone or in combination, play a role in germination traits and development of *C. canadensis*, while pH had an influence only under interaction with certain nutrients (Figs. 1, 2, 3, 4, 5). Below, we will present a discussion based on the synergistic effect, no-effect, or antagonistic effect that nutrients and pH had on seed germination and the development of *C. canadensis.* We will discuss also the finding implications on weed management at field conditions.

### Nutrient as factor conditioning horseweed germination and seedling development

In the literature, nitrogen is one of the most studied nutrients that affect the horseweed germination process^31^^,^^32^^,^^33^, while studies on other nutrients are scarce. Here, the findings clearly show that the exposure of horseweed seeds to different nutrients affected the final germination percentage and initial development of *C. canadensis* (Figs. 1, 2, 3, 4, 5). Furthermore, a large range of nutrient combinations had no-effect on the germination traits and development of horseweed. Phosphorus had an antagonistic effect on the following parameters: FGP, GSI, SL (at pH 4.8), and RSR for both pH (4.8 and 6.5). The P alone seems to inhibit germination and early horseweed development, probably due to the imbalance of nutrients in the seeds^34^. However, P exposure in combination with K or Ca and Mg allowed a synergistic effect on *C. canadensis* seedling development (SL and RL). Our study evidenced that P inhibited germination process, but the seedling development was busted with the P presence in combination with other nutrients.

The exposure of certain nutrients such as N, or metals such as Al, promotes a competition of nutrients out and inside the seeds, degrading the cell’ seeds^34^. However, when salt stress (Cl^-^) was tested with different cations (Mg, Na, and Ca), the germination traits were also affected^20,21,35^. As horseweed seeds do not undergo dormancy^33^, the germination process starts immediately when seeds fall out on topsoil in any crop season^14,36^. Thus, the conditions surrounding the seeds become determinants to germination trait responses^37^. In addition, the same nutrient may act equally or differently on the *C. canadensis* germination and seedling development processes depending on the pH, as clearly evidenced here for P alone or in combination with others ions (Figs. 1, 2, 3, 4, 5). As nutrients and pH conditions affected the horseweed germination, several physiological mechanisms may be involved as follow: seed nutrient balance, physiological seed deterioration, osmotic stress, and nutrient toxicity^34,38^. However, these mechanisms need to be better explored in future studies to answer how nutrients act in the germination process. The answer requires an understanding of the seed germination response beyond temperature and moisture substrate conditions, considering ion competition and pH conditions that influence seed deterioration/germination^34^. It is known that chemical osmotic effects from nutrient exposure easily led to changes in metabolic and physiological seed behavior^14^. Woodstock et al.^39^ found that seed quality was associated with seed capacity for ion releases, as a response to adverse conditions. The mechanism is then associated with the physical integrity of seed membranes, which ensures high germination potential but may be altered by nutrient availability of the growing media.

In our study, seeds exposed to different ions varied the final germination percentage and seedling development, sometimes having no-effect on the germination and sometimes decreasing when compared to the control (distilled water only). Phosphorus exposure presented an antagonist effect on germination probably because the ion balance affected other mandatory ions of physiological importance, such as Zn inside the seed, which is an important nutrient for the seed’s germination process^40^. In contrast to the P, other nutrients alone or combined with Ca, Mg and K presented no-effect on germination. Yamashita and Guimarães (2011a) found Al at 1.5 cmolc Kg of soil (low Al contents) diminishes *C. canadensis* germination by 24%. In addition, calcium chlorate affects *C. canadensis* germination and GSI in concentrations higher than 6 cmolc L^-1^ and 2 cmolc L^-1^, respectively^20^. These studies suggest that seed responses depend on the ion companion in the salt molecule, as reinforced here. Finally, our study provided a large range of nutrients which produced different responses on germination and development of horseweed species.

The synergistic effect on RSR was observed when several nutrients were combined at low pH. When a large nutrient availability (P, K, Ca, Mg) is offered to the horseweed the plant allocates nutrients in the root system. Kuchenbuch & Jung (1988) stated that a non-restrictive nutrient condition reaches a high RSR – high root system comparatively with shoots. Thus, adequate soil/substrate chemical conditions tents to increase RSR. On the other hand, P availability had an antagonistic effect on RSR. It seems that P availability promoted a shoot allocation comparatively to the root allocation^41^.

Even under unfavorable edaphic conditions, horseweed development was assured when a large variety of nutrient was available except for P, that had an antagonistic effect. It reinforces that horseweed is able for surviving in adverse and eutrophic soil conditions, as reported by Concenço and Concenço (2016)^10^.

### Acidity as factor conditioning horseweed germination and seedling development

Few studies were produced on horseweed testing chemical conditions (nutrients vs pH) on germination process^20,21,40^. The literature shows that horseweed germination is favored by neutral pH conditions even under saline conditions^18,20,21^. Here, two pH levels were tested, which pH affected only SL values as a single factor. However, pH presented interaction with some nutrients alone or in combination (K, Ca, P, and Mg). Low pH (4.8) associated with Ca presented higher values for FGP and GSI. Furthermore, seedling elongation seemed to be benefited at pH 4.8, regardless of the nutrient combination studied. At pH (6.5), PMg nutrients combination presented higher values for FGP and RL, SL and RSR than a pH 4.8. pH 6.5 associated with the nutrients reached high values for the majority parameter evaluated, excepting SL. In the control treatment (only with distilled water), GSI and RSR values were higher at 6.5 than at pH 4.8 while for SL was the value was higher at pH 4.8.

In the literature, different pH levels (4.7, 5.7, 6.7, and 7.7) and nitrogen concentration in eight species at Spain^35^ (covered the following families: Fabaceae, Onagraceae, Apiaceae, Poaceae, and Polygonaceae) were tested and it was found that the germination process is greatly dependent of N amounts and forms, but is not associated with pH levels. The pH influences the germination process but depends on the species^29^. Laghmouchi et al. (2017)^3125^, did not find evidence of pH effects on the seed germination of *Origanum compactum* and *Capsella bursa-pastoris* in a large range of pH. However, Gentili et al. (2018)^32^ studying an Asteraceae species (*Ambrosia artemisiifolia*) suggests that low soil pH (5.0) affects positively the growth and development while neutral pH limited it. Furthermore, contrasting pH effects have been described on germination dependence on the species studied^31,42^. In this sense, the germination of many species responds rather to salt stress and nutrient availability than pH levels^43^.

Our findings indicate that pH is not a critical factor for germination traits when taken into account as an isolated factor, but in combination with nutrients assumes a relevant role. Thus, this study provides good knowledge on the effect of ions on the *C. canadensis* germination and seedling development, which allows us to plan adequate soil management to reduce the weed pressure in croplands since the knowledge in the literature is scarce^14,18,22,36^.

### Findings implication for horseweed management on field conditions

Here, it was demonstrated that different ions affected the germination traits of *C. canadensis*. Light, temperature, and water availability were controlled in our laboratory’s experiment, varying only nutrients availability and pH conditions. In addition, the doses used for the treatments are compatible with the doses of Ca, Mg, P, and K normally receipt as fertilizers on topsoil from no-till system cropped with soybean. Thus, we expected that the findings found here can be a subsidiary under the field situation, concerning only the pH and nutrients availability.

In this sense, the P nutrient stood out as the most important nutrient in germination trait and seedling growth, and secondly, the pH was determinant mainly in combination with nutrients. Despite this study has been conducted under controlled conditions, our results showed that P alone or in combination with Ca inhibited horseweed seed germination percentage around 7.28 and 1.64 times compared to the control. However, after seed germination, the combination of P with other nutrients (eutrophic conditions) had a synergistic effect on seedling development. In addition, P alone had a reduction of 4.28 times in GSI.

From the agronomic perspective, our study suggests that topsoil chemical conditions should be taken into account to develop effective and integrated alternatives for *C. canadensis* control. Tudela-Isanta et al. (2018) stated that low pH promoting increases in soil aluminum contents, but pH is micro factor stress controlling seed germination niche in habitat management^44^. It is well-known that liming regulates soil acidity and by consequence species ability for land invasion^27^. Thus, the occurrence, distribution, and hazardousness of weed species may be handling partially by soil chemical factors^37^.

Our approach became mandatory for horseweed control in no-till systems because (i) the ecological and physiological species characteristics favor their occurrence in croplands^15^, including high soil seed bank^8^ (ii) the low chemical horseweed control efficiency due to the herbicide resistance mechanisms of *C. canadensis*^4,12,13^ and, (iii) the low quality of a major no-till system practiced, including low crop residue^3^, topsoil P and K fertilization, which produces an intense soil eutrophication^28^.

As seed germination is one of the main mechanisms of alien species to invade and establish on agricultural areas^43^, handling ecological aspects of this mechanism may aid plant management^23^. The final germination percentage of horseweed varied from 6 to 35.5% following the nutrient available. This behavior may indicate the amplitude in the control effectiveness associated with fertilizer management in no-till system. In general, the species had a high capacity for land invasion when the seeds find favorable conditions (adequate temperature, moisture, and chemical environment). Although the species produce large quantities of seeds that are easily disseminated^15^, an adequate chemical environment may contribute to diminish the weed establishment. In this sense, the fertilization method commonly used in no-till system areas for furnish Ca and Mg (under lime product), P and K (under triple superphosphate and potassium chloride) transformed the topsoil in eutrophic condition^28^. This information may answer why in areas under no-till system in Brazil there is a high abundance of this weed species^4^. Thus, the manner of fertilization and liming should be revisited because it may favor the occurrence and establishment of horseweed in this system.

Although our results were obtained in laboratory conditions, they suggest that the topsoil with low nutrient contents and low pH level consists in an adequate edaphic condition for reaching low germination percentage and speed germination of *C. canadensis*. Furthermore, we state that adequate fertilization in no-till areas may improve crop nutrition efficiency and may assist in effective weed control, reducing pesticide use, and improving environmental quality. In addition, a good NT system must be properly conducted, observing the adequate straw quantity and quality, a good crop rotation to minimizes soil disturbance diminishing seed quantities in the soil seed bank, which follows recommendations of several authors^2,39,45^. These ideas are not novelty; classical studies in phytosociology, in which species ecology and edaphic conditions control the occurrence of species in environments, are readily available^6,8,23,24,27,39,44^.

Finally, our results contribute to an integrative strategy in weed management, in which nutrient management may be used to diminishing horseweed germination in no-till areas that present a high germination percentage^8,45^. Thus, we expected that low horseweed germination results in low horseweed occurrence and pressure on crops, reducing environmental pressure^9^.

## Conclusions and implications

This work corroborates the hypothesis in which the seed exposure to nutrients, alone or in combination, and different pH levels (4.8 and 6.5) are important factors controlling seed germination traits and seedling development of horseweed seeds. Regarding the nutrient effect, the phosphorus alone has an antagonistic effect on the final germination percentage of horseweed in both pH tested. However, nutrient richness, including P, surrounding horseweed seeds shows a synergistic effect the seedling development (root length and shoot length). Medium acidity (pH level) is a secondary factor influencing the germination traits, but pH 4.8 promotes horseweed seedling development (increasing shoot length). The pH is a preponderant factor when in interaction with certain nutrients, presenting a high germination speed index at pH 6.5 in the control treatment and associated with K and at pH 4.8, when in association with Ca, KMg, and PMg.

These findings suggest that chemical topsoil conditions favor or inhibit seed germination traits and seedling development of horseweed species. Thus, chemical edaphic status aid to explain the weed occurrence in agricultural areas. The implication of our findings fills a scientific gap and fits as a useful approach to integrate future strategies of weed control, improving the substantiality in modern agriculture.

## Materials and methods

### Local of study

The present study was carried out under controlled conditions at the University of Passo Fundo, State of Rio Grande do Sul, Brazil and complies with local and national regulations. The experiment used Canadian horseweed seeds (*Conyza canadensis* (L.) Cronquist var. canadensis) bought from a commercial farmer and distributer (Agrocosmos 13,165–970, São Paulo, Engenheiro Coelho, Brazil). The seeds were originated from the owner production (the year of 2018) at São Paulo State (tropical climate regime). The seeds were managed at the Seed Analysis Laboratory by the Passo Fundo University, with Accreditation in the ISO/IEC 17,025—general requirements for the competence of testing and calibration laboratories, under the responsibility of Dr. Nadia Canali Lângaro. An aliquot of seeds used in this study (~ 10 g) was deposited in an herbarium of the Program of Post-graduation in Agronomy of the University of Passo Fundo (504–302,224—2019), for public access. The seeds were then submitted to the germination process in different nutrient solutions, in an environmentally controlled chamber.

### Experimental design and assay conditions

We used 14 nutrient solutions containing P, K, Ca, and Mg, alone and in combination^46^. These elements were chosen because they are normally used in topsoil liming and fertilization under no-till crop system. The nutrient quantities used in topsoil follow in decreasing order Ca, Mg, K, and P. Each solution was divided into two aliquots: the pH of the first aliquot was adjusted to 4.8 with chloric acid (HCl), and the pH of the second aliquot was adjusted to 6.5 with sodium hydroxide (NaOH). Thus, the trial was divided into two parts according to pH, with 14 treatments each, plus the control (distilled water) (Table 2). We assume that pH levels were maintained or had low variation during the assay. Additionally, all material used in the experiment was sterilized with ethanol (70%, v:v).

We prepared nutrient solutions according to recommended lime/fertilizer doses used in the field for soybean crop^47^. In the field soybean crop, the fertilizer formulas and doses commonly used are: potassium chloride (KCl) at the dose of 80 kg/ha, or 25.6 kg of K per ha, and triple superphosphate at the dose of 40 kg/ha, or 35.2 kg of P per ha. For Ca and Mg, the formulas and doses were based on the lime practice at 2 ton/ha of dolomitic limestone applied on the topsoil. Thus, the doses per hectare of Ca is 992 kg, while Mg is 592.2 kg. A more detailed explanation of the doses for each treatment is given in Table 2.

In the laboratory, the nutrient solutions were prepared using distilled water combined with phosphoric acid (H_3_PO_4_) as a P source, potassium sulfate (K_2_SO_4_) as a K source, calcium hydroxide (Ca(OH)_2_) as a Ca source, and magnesium oxide (MgO) as a source of Mg. High chemical purity reagents were used (P.A. Merck Co). The doses were calculated taken into account the surface area of plastic boxes for germination test—gerbox (10 cm × 10 cm × 3.5 cm).

Germitest paper (J. Prolab, São José dos Pinhais, PR, Brazil) placed in gerbox plastic boxes received 10 mL of nutrient solutions and 50 horseweed seeds per replicate. These composed the experimental units, which were incubated, in quadruplicate, in controlled climate chambers (volume capacity: 354 L) for 15 days at 24 °C (± 0.5 °C) in light 12/12-h photoperiod (Light characteristics: four OL T8, 8 W, 6500 k led lamps). The boxes were covered using plastic film to maintain the similar moisture during the assay.

### Plant analyses

Over 10 days, the gerbox treatments were daily monitored to estimate the final germination percentage (FGP) and germination speed index (GSI). The seeds with root length over 2 mm were considered as a seed that accomplished their germination process and were recorded daily. The experiment was conducted based on the protocol proposed by the official seed analysis from the Brazilian government^48^.

The final germination percentage was calculated following Eq. (1), and is expressed as a percentage. The germination speed index (GSI) was evaluated using Eq. (2).

1 $${\text{FGP }} = \, \left( {\sum {\text{n}}_{{1}} /{\text{NS}}_{{\text{i}}} } \right) \times {1}00$$

where FGP is the total seeds that accomplished their germination process (Ʃn_1_) in relation to the total of seeds used in the assay (NS_i_);

2 $${\text{GSI }} = {\text{ G}}_{{1}} /{\text{N}}_{{1}} + {\text{G}}_{{1}} /{\text{N}}_{{2}} + \cdots {\text{G}}_{{\text{n}}} /{\text{N}}_{{\text{n}}}$$

where, G_1_, G_2_, G_n_ are the number of seeds that accomplished their germination process for the day and N_1_, N_2_, N_n_ are the number of days after the assay start.

Seedling shoot length (SL) and root length (RL) were evaluated at the end of the experiment (15 days after the start of the assay). The root:shoot ratio values were calculated using data of RL and SL.

### Data analysis

A two-way analysis of variance, ANOVA (*P* < 0.05) was performed in a bi-factorial randomized design (14 nutrient treatments + control *vs* 2 pH) with post-hoc Tukey’s range test (*P* < 0.05) where all means of treatments were compared together^49^.

Statistical analysis was performed on R-Studio (version 3.6.1, 2019-07-05), an open-source software, and graphics were created on Sigmaplot (version 13). The normality of the distribution of residuals was tested by a *P*-value < 0.05 in a Shapiro–Wilk test, and the homogeneity of variances by a *P-*value < 0.05 in a Bartlett test. The Box-cox technique was employed to transform residuals from a non-normal distribution to a normal distribution. Principal component analysis (PCA) was performed in the dataset at *P* < 0.1, for each pH level separately, using open-source software Past (version 3.23). The principal components (axes PC1 and PC2) explain the magnitudes in the percentage of data variability.

The data is presented to show which nutrients (alone or in combination) present a synergic, no effect, or antagonist effect compared with the control (distilled water). Thus, the synergistic effect is when average values were statistically higher than control treatment; no-effect means that there was no statistical difference, and antagonistic effect means low values compared with the control treatment.

### Ethics approval

This manuscript consists of original research that has not been published before and is not currently being considered for publication elsewhere. Besides, the manuscript has been read and approved by all named authors.

### Consent to participate and for publication

The authors consent for the participation in this publication.

**Table 1:** Summary of two—way analysis of variance (F and p—values) of germination traits of *C. canadensis* (final germination percentage; germination speed index) the seedlings traits (seedling and root length, root:shoot ratio) of nutrients and pH levels.

Source of variation	df	FGP			GSI		SL		RL		RSR		*n* =
		F-value	p-value		F-value	p-value	F-value	p-value	F-value	p-value	F-value	p-value	
F1: ion	14	20.20	0.001*		8.47	0.001*	52.65	0.001*	8.01	0.001*	8.22	0.001*	60
F2: pH	1	1.999	0.160 ns		0.006	0.938 ns	185.17	0.001*	0.152	0.69 ns	19.05	0.001*	60
F1 × F2	14	1.594	0.096 ns		3.055	0.001*	48.455	0.001*	3.286	0.001*	4.08	0.001*	120
CV, %		24.1			26.2		31.0		44.9		37.9		

*Significant at the p value indicated as error probability level. ns considered not statistically significant at the probability level.

df, degree of freedom; n, number of experimental units (treatments × replications); CV, coefficient of variation; FGP, final germination percentage, %; GSI, germination speed index, arbitrary unit; SL, seedling length in their aerial part, mm; RL, root length, mm; RSR, Root:shoot ratio, arbitrary unit.

**Table 2:** Concentration of nutrient alone or in combination in the solutions used as treatment.

Nutrients alone or in combination	Nutrient concentration (mg gerbox^−1^)			
	P	K	Ca	Mg
P	35.2			
K		25.6		
Ca			992	
Mg				592
PK	35.2	25.6		
PCa	35.2		992	
PMg	35.2			592
KCa		25.6	992	
KMg		25.6		592
CaMg			992	592
PKCa	35.2	25.6	992	
KCaMg		25.6	992	592
PCaMg	35.2		992	592
PKCaMg	35.2	25.6	992	592
Control	Distilled water only			

Nutrient source: K_2_SO_4_ 0.000654 M; H_3_PO_4_ 0.00111 M; Ca(OH)_2_ 0.0496 M; MgO 0.0493 M.

## Supplementary Information

Supplementary Information.

## Data Availability

We supplied all data. All data used in this study were included in the Supplementary Information File.

## Abbreviations

*Conyza canadensis* (L.) Cronquist var. canadensis: *C. canadensis*
FGP: Final germination percentage
GSI: Germination speed index
RL: Root length
SL: Seedling length in the aerial part
RSR root: Shoot ratio
P: Phosphorus
K: Potassium
Ca: Calcium
Mg: Magnesium
NT: No-till system
