# Supplementary material for: Nutrient availability and pH level affect germination traits and seedling development of Conyza canadensis
Source: Sci Rep. 2021 Aug 2;11:15607. doi: 10.1038/s41598-021-95164-7 (PMC8329304; doi:10.1038/s41598-021-95164-7)
Supplement: Supplementary file 1 — Supplementary Information. [file 41598_2021_95164_MOESM1_ESM.docx]

**Complementary data**

Post-hoc Tukey (*P<*0.05) test of germination traits of *C. canadensis* (germination percentage; germination speed index) the seedling development (seedling and root length. root:shoot ratio) of nutrients and pH levels. Lower case compares nutrients in the column; upper case compared the same nutrient between two pH in the line.

| Final germination porcentage - FGP | | | | | | | | |
| --- | --- | --- | --- | --- | --- | --- | --- | --- |
|  | pH 4.8 |  | Tukey´s test | | pH 6.5 |  | Tukey´s test | |
|  | Mean | SD |  |  | Mean | SD |  |  |
| K | 31.00 | 5.29 | a |  | 36 | 12.1 | a |  |
| PKCaMg | 29.00 | 3.46 | ab |  | 28.5 | 5.26 | ab |  |
| KCa | 29.00 | 2.58 | ab |  | 28.5 | 9.43 | ab |  |
| KCaMg | 22.00 | 5.16 | ab |  | 28 | 5.16 | ab |  |
| KMg | 32.50 | 4.43 | a |  | 28 | 4.00 | ab |  |
| Mg | 31.00 | 5.03 | a |  | 28 | 4.32 | ab |  |
| CaMg | 29.00 | 3.83 | ab |  | 27.5 | 5.74 | ab |  |
| Control | 25.50 | 3.42 | ab |  | 25.5 | 6.61 | ab |  |
| PKCa | 25.50 | 6.61 | ab |  | 24.5 | 5.26 | ab |  |
| PMg | 16.00 | 6.32 | bc |  | 24.5 | 6.19 | ab |  |
| PCaMg | 24.00 | 8.33 | ab |  | 22 | 1.63 | ab |  |
| PK | 29.00 | 6.63 | ab |  | 21 | 3.83 | ab |  |
| Ca | 29.00 | 8.41 | ab |  | 17.5 | 4.73 | bc |  |
| PCa | 23.00 | 9.59 | ab |  | 15.5 | 5.74 | bc |  |
| P | 6.00 | 2.83 | c |  | 3.5 | 2.52 | c |  |

| Germination speed index - GSI | | | | | | | | |
| --- | --- | --- | --- | --- | --- | --- | --- | --- |
|  | pH 4.8 |  | Tukey´s test | | pH 6.5 |  | Tukey´s test | |
|  | Mean | SD |  |  | Mean | SD |  |  |
| KMg | 4.69 | 0.58 | a |  | 3.90 | 0.42 | ab |  |
| KCa | 4.45 | 0.35 | a |  | 3.73 | 1.45 | ab |  |
| CaMg | 4.43 | 0.58 | a |  | 3.92 | 0.73 | ab |  |
| PKCaMg | 4.36 | 0.59 | a |  | 4.12 | 1.16 | ab |  |
| PKCa | 3.99 | 1.02 | ab |  | 3.64 | 0.93 | ab |  |
| Mg | 3.96 | 0.55 | ab |  | 3.92 | 0.68 | ab |  |
| Ca | 3.84 | 0.81 | ab | A | 2.50 | 0.74 | bc | B |
| PK | 3.73 | 0.96 | ab |  | 3.07 | 0.53 | ab |  |
| PCaMg | 3.68 | 1.21 | ab |  | 3.29 | 0.24 | ab |  |
| PCa | 3.35 | 1.49 | ab |  | 2.11 | 0.46 | bc |  |
| K | 2.90 | 0.42 | ab | B | 5.16 | 1.60 | a | A |
| KCaMg | 2.86 | 0.56 | ab |  | 3.71 | 0.94 | ab |  |
| Control | 2.57 | 0.40 | abc | B | 3.90 | 1.75 | ab | A |
| PMg | 2.01 | 0.92 | bc | B | 4.08 | 1.28 | ab | A |
| P | 0.61 | 0.28 | c |  | 0.56 | 0.56 | c |  |

Cont….

| Root Length - RL | | | | | | | | |
| --- | --- | --- | --- | --- | --- | --- | --- | --- |
|  | pH 4.8 |  | Tukey´s test | | pH 6.5 |  | Tukey´s test | |
|  | Mean | SD |  |  | Mean | SD |  |  |
| PKCaMg | 4.32 | 2.29 | a |  | 2.82 | 0.85 | abc | A |
| KCa | 3.47 | 0.25 | ab | A | 1.04 | 0.07 | bcd | B |
| KMg | 3.05 | 0.76 | ab | B | 5.23 | 2.93 | a | A |
| CaMg | 3.84 | 0.48 | ab |  | 3.67 | 0.65 | ab |  |
| PKCa | 3.32 | 1.12 | ab |  | 3.07 | 1.28 | abc |  |
| KCaMg | 3.04 | 1.38 | ab |  | 2.99 | 1.55 | abc |  |
| PK | 2.76 | 0.50 | abc |  | 2.59 | 0.88 | abcd |  |
| K | 1.78 | 0.31 | abcd |  | 2.36 | 0.47 | bcd |  |
| Ca | 2.08 | 0.91 | abcd |  | 0.70 | 0.19 | cd |  |
| Mg | 2.17 | 0.24 | abcd |  | 2.74 | 0.78 | abc |  |
| PCaMg | 2.39 | 0.65 | abcd |  | 1.84 | 0.36 | bcd |  |
| PCa | 1.52 | 1.01 | bcd | B | 3.24 | 2.06 | abc | A |
| Control | 1.28 | 0.45 | bcd |  | 1.09 | 0.22 | bcd |  |
| PMg | 0.37 | 0.43 | cd | B | 3.15 | 1.56 | abc | A |
| P | 0.00 | 0.00 | d |  | 0.00 | 0.00 | d |  |

| Seedling Length - SL | | | | | | | | |
| --- | --- | --- | --- | --- | --- | --- | --- | --- |
|  | pH 4.8 |  | Tukey´s test | | pH 6.5 |  | Tukey´s test | |
|  | Mean | SD |  |  | Mean | SD |  |  |
| K | 8.65 | 1.04 | a | A | 1.25 | 0.10 | ab | B |
| PK | 9.34 | 1.49 | a | A | 1.28 | 0.34 | ab | B |
| Control | 5.35 | 1.10 | b | A | 0.67 | 0.15 | ab | B |
| KMg | 2.30 | 1.67 | c |  | 1.90 | 0.34 | a |  |
| PKCaMg | 1.79 | 0.28 | c |  | 1.43 | 0.40 | a |  |
| CaMg | 1.76 | 0.34 | c |  | 1.59 | 0.24 | a |  |
| KCa | 1.63 | 0.25 | cd | A | 0.77 | 0.12 | ab | B |
| KCaMg | 1.54 | 0.51 | cd |  | 1.36 | 0.31 | ab |  |
| PKCa | 1.47 | 0.30 | cd |  | 1.40 | 0.52 | ab |  |
| PCaMg | 1.36 | 0.16 | cde |  | 1.16 | 0.25 | ab |  |
| Mg | 1.27 | 0.19 | cde |  | 1.29 | 0.09 | ab |  |
| Ca | 1.00 | 0.35 | cde |  | 0.52 | 0.01 | ab |  |
| PCa | 1.00 | 0.71 | cde |  | 1.40 | 0.52 | ab |  |
| PMg | 0.27 | 0.32 | de | B | 1.21 | 0.31 | ab | A |
| P | 0.00 | 0.00 | e |  | 0.00 | 0.00 | b |  |

Cont.

| Root Length: Shoot Length ratio - RSR | | | | | | | | |
| --- | --- | --- | --- | --- | --- | --- | --- | --- |
|  | pH 4.8 |  | Tukey´s test | | pH 6.5 |  | Tukey´s test | |
|  | Mean | SD |  |  | Mean | SD |  |  |
| PKCaMg | 2.33 | 0.85 | a |  | 2.02 | 0.58 | a |  |
| CaMg | 2.26 | 0.63 | a |  | 2.36 | 0.60 | a |  |
| PKCa | 2.23 | 0.47 | a |  | 2.21 | 0.50 | a |  |
| KCa | 2.15 | 0.16 | ab |  | 1.38 | 0.16 | ab |  |
| KCaMg | 2.07 | 1.05 | ab |  | 2.17 | 0.88 | a |  |
| Ca | 2.02 | 0.29 | ab |  | 1.35 | 0.37 | ab |  |
| Mg | 1.74 | 0.38 | abc |  | 2.11 | 0.49 | a |  |
| PCaMg | 1.74 | 0.36 | abc |  | 1.60 | 0.19 | a |  |
| KMg | 1.72 | 0.90 | abcd | B | 2.84 | 1.79 | a | A |
| PCa | 1.17 | 0.82 | abcde | B | 2.20 | 0.62 | a | A |
| PMg | 0.68 | 0.79 | bcde | B | 2.48 | 0.70 | a | A |
| PK | 0.31 | 0.10 | cde | B | 2.01 | 0.41 | a | A |
| Control | 0.24 | 0.03 | cde | B | 1.67 | 0.37 | a | A |
| K | 0.21 | 0.04 | de | B | 1.90 | 0.35 | a | A |
| P | 0 | 0 | e |  | 0 | 0 | b |  |
